# Intradermal administration of IL-33 induces allergic airway inflammation

**DOI:** 10.1038/s41598-017-01863-5

**Published:** 2017-05-10

**Authors:** Hongwei Han, Steven F. Ziegler

**Affiliations:** 1 Immunology Program, Benaroya Research Institute, Seattle Washington, 98101 USA; 2 Department of Immunology, University of Washington School of Medicine, Seattle Washington, 98195 USA

## Abstract

Approximately half of all atopic dermatitis (AD) patients subsequently develop asthma, particularly those with severe AD. This association, suggesting a role for AD as an entry point for subsequent allergic disease, is a phenomenon known as the “atopic march”. While the underlying cause of the atopic march remains unknown, recent evidence suggests that epithelial cell (EC)-derived cytokines play a major role. We showed that mice exposed to antigen through the skin, in the presence of IL-33, developed antigen-specific airway inflammation when later challenged in the lung. IL-33 signaling was dispensable during effector/challenge phase. These data reveal critical roles for IL-33 in the “atopic march” and will offer a new therapeutic target in the treatment and prevention of allergic asthma.

## Introduction

Atopic dermatitis (AD) is the most common chronic inflammatory skin disease in the United States, affecting an estimated 10 to 20 percent of infants and young children^1, 2^. Recent studies suggest that the skin may be an important site for systemic sensitization to allergens, leading to the development of allergic inflammatory responses at other sites, a phenomenon referred to as the “atopic march”. It has been recognized that genetic pre-disposition^3, 4^ as well as environmental factors potentially underlie causes. Mutations within the AD susceptibility genes, such as filaggrin (*Flg*) and *SPINK5*, are also associated with an increased risk of asthma^5–11^. Biological mechanisms such as sensitization of innate and adaptive immune responses, skin barrier defects as well as epigenetic changes have been proposed to elucidate the mechanism underlying the “atopic march”. In animal models, epicutaneous allergen exposure induces allergic airway inflammation after re-challenge with the same antigen in the lung^12–18^. Thus, the early immunological events and factors involved in skin sensitization to allergens may be an important focal point in understanding the development of not only AD but also other atopic diseases.

IL-33, an IL-1 family cytokine, is associated with multiple allergic disorders and is thought to induce allergic inflammation by activating mast cells, inducing Th2 responses, supporting IgE production and eliciting the population expansion of group 2 innate lymphoid cells (ILC2s)^19, 20^. IL-33 and the receptor ST2 are expressed in skin and is elevated in humans and mouse models of AD^20–22^, and IL-33 is increased in the submucosa of patients with steroid-resistant asthma^23^. Increased expression of IL-33 and ST2 has been observed in human AD skin after house dust mite (HDM) or staphylococcal enterotoxin B (SEB) exposure. Combined stimulation with tumor necrosis factor (TNF)-α and IFN-γ leads to an increase in IL-33 protein expression in primary human dermal fibroblasts and keratinocytes^21^. Furthermore, IL-33 may also affect barrier function by downregulating filaggrin in subconfluent and differentiated monolayer keratinocytes and skin biopsies^24^. In genetic studies, single nucleotide polymorphisms (SNPs) in the distal promoter of the ST2 gene locus (*Il1rl1*) are significantly linked to AD prevalence, suggesting the IL-33-ST2 pathway might be a risk factor for AD^19^. Several independent genome wide association studies (GWAS) have also implicated the IL-33 and ST2/IL1RL1 (IL-33 receptor) genes as asthma susceptibility loci^25, 26^.

Mouse models have extended our understanding of the requirements for IL-33 signaling in allergic diseases, showing that IL-33 expression increases in the lung during both the early and chronic phases of airborne allergen exposure, and that airway inflammation is significantly attenuated in *Il1rl1-*deficient mice^27^. Furthermore, local delivery of IL-33 is sufficient to drive inflammation in the skin and lung^28^. In addition, although it is controversial, ectopic expression of IL-33 in keratinocytes could induce AD-like symptoms in mice^28^. As AD is closely associated with asthma, IL-33 could also be secondarily involved in skin-mediated asthma development.

Using a mouse model of atopic march, we demonstrate that epicutaneous sensitization to model antigen OVA in the presence of excess IL-33 leads to Th2-driven allergic responses following antigen exposure in the lung. In this model skin IL-33 is sufficient for the development of antigen-induced airway allergy. However, IL-33 was not required during challenge in the lungs, indicating that targeting IL-33 signaling pathway during AD development might offer a new therapeutic insight in the prevention of asthma.

## Results

### Intradermal injection of IL-33 results in a Th2 phenotype and skin inflammation

**Figure 1:**
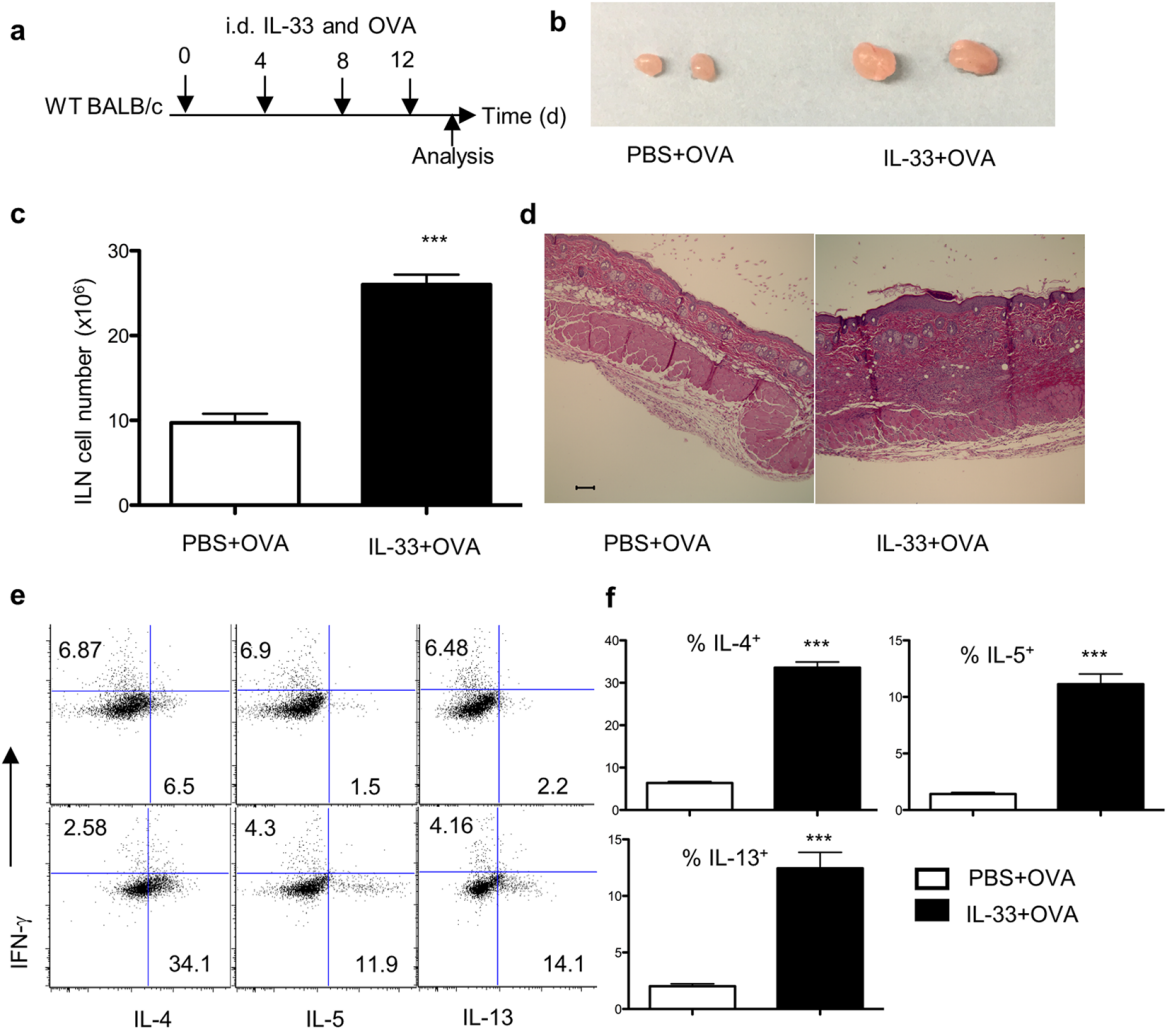
Injection of IL-33 induces a Th2 cell phenotype and skin inflammation. (**a**) Experimental protocol. Mice were analyzed on day 15. (**b**) Representative inguinal lymph node (ILN) from PBS + OVA (control) and IL-33 + OVA treated mice. (**c**) ILN cellularity. (**d**) H&E-stained section of skin. Bar, 100 µm. (**e**) Intracellular cytokine staining of ILN cells. Plots are gated on CD4^+^CD44^hi^ cells. Upper, PBS + OVA; lower, IL-33 + OVA. Data were pooled from two independent experiments (n = 7). Error bars indicate the mean ± SD. ***p ≤ 0.001.

We first developed a model in which recombinant IL-33 protein was delivered into the intradermal space. Wild-type (WT) BALB/c mice were injected intradermally (i.d.) with recombinant, carrier-free IL-33 plus a model antigen, ovalbumin (OVA), on their backs four times over a 12-day period (Fig. 1a). Mice receiving IL-33 exhibited an increase in draining lymph node (DLN) cellularity, epidermal thickening and dermal infiltration (Fig. 1b–d). Re-stimulated DLN cells from mice injected with IL-33 produced increased amounts of IL-4, IL-5 and IL-13 (Fig. 1e).

### Intradermal administration of IL-33 promotes antigen-induced allergic airway inflammation

**Figure 2:**
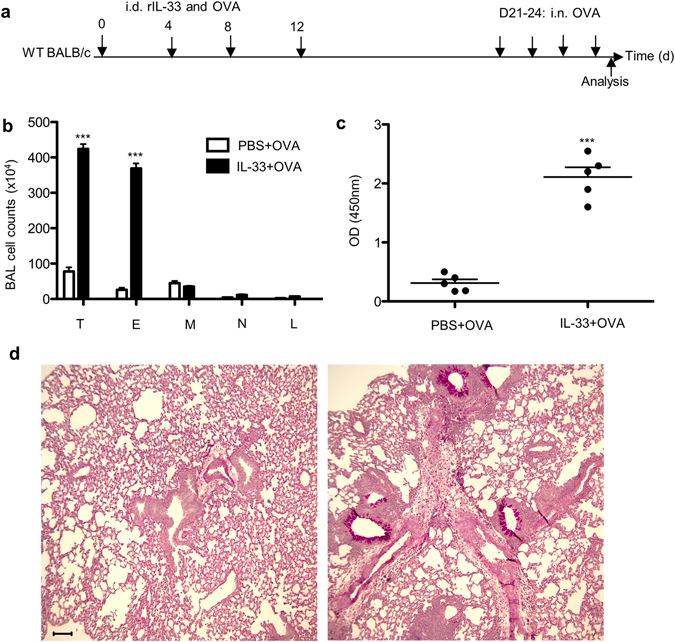
Intradermal administration of IL-33 aggravates airway inflammation and mucus secretion in experimental asthma. (**a**) Experimental protocol. (**b**) Cell counts in the BAL fluid. The significance between two groups was determined by two-tailed Student’s t test. (**c**) OVA-specific IgE on Day 25. (**d**) Representative lung tissue cross-sections stained with PAS to visualize mucus-producing goblet cells and airway inflammation. Left, PBS + OVA; Right, IL-33 + OVA. Bar, 100 µm. T, total cells; E, eosinophils; M, macrophages; N, Neutrophils; L, lymphocytes. Data are representative of two independent experiments with five mice per group. Error bars indicate the mean ± SD. ***p ≤ 0.001.

Epidemiological studies have shown that children with AD in infancy or early childhood are likely to also develop asthma^3^. Given that the skin epithelium is a major source of IL-33, a cytokine that can drive Th2-type responses in both the skin and lung, IL-33 is a likely candidate linking AD and asthma. We established a mouse atopic model in which wild-type BALB/c mice were sensitized with recombinant IL-33 plus OVA, followed by intranasal (i.n.) OVA challenges (Fig. 2a). In this model, IL-33-treated mice exhibited an asthmatic phenotype evidenced by increases in bronchial alveolar lavage (BAL) cellularity and OVA-specific IgE levels compared to control mice not exposed to IL-33 (Fig. 2b,c). In addition, lungs from these mice also showed enhanced goblet cell metaplasia and mucus overproduction by periodic acid Schiff (PAS) staining (Fig. 2d). Re-stimulated mediastinal lymph node (MedLN) cells from mice sensitized with IL-33 produced increased amounts of IL-4, IL-5 and IL-13 (Supplementary Fig. 1). Taken together, these data indicate that excess IL-33 activity in the skin can convert an otherwise harmless antigen exposure into allergen sensitization that leads to subsequent pulmonary reactions to allergen exposure.

**Figure 3:**
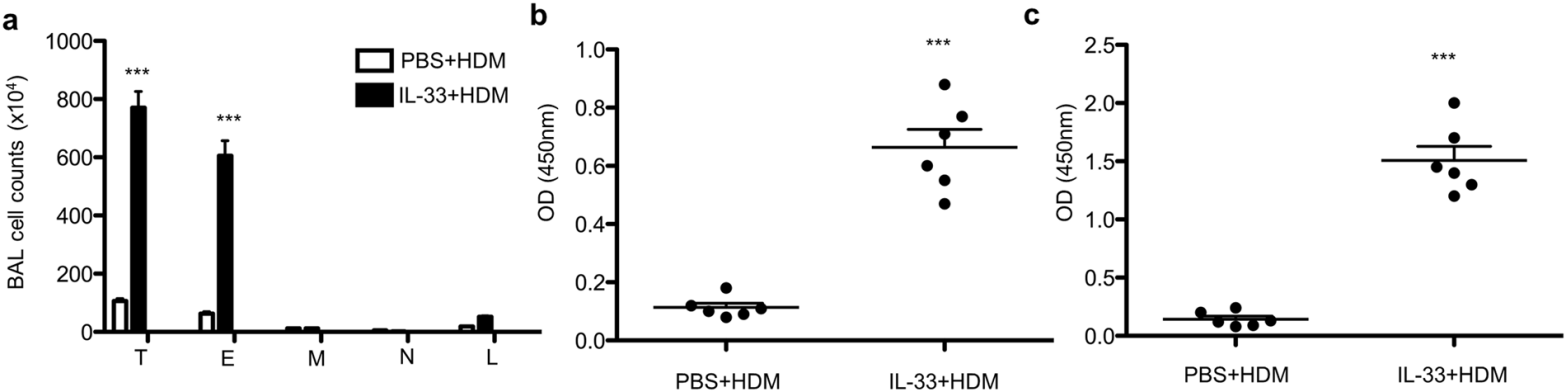
Skin sensitization with HDM + IL-33 induces airway inflammation. (**a**) Cell counts in the BAL fluid. (**b**) HDM-specific IgE in BAL fluid. (**c**) HDM-specific IgE in serum. T, total cells; E, eosinophils; M, macrophages; N, Neutrophils; L, lymphocytes. Data were pooled from two independent experiments (n = 6). Error bars indicate the mean ± SD. ***p ≤ 0.001.

House dust mite (HDM) allergen is a common and clinically relevant aeroallergen that associate with exacerbation of AD^29^. We next sensitized mice intradermally with HDM and examined airway inflammation upon i.n. challenge. We observed a similar pattern of disease in mice when HDM antigen was used (Fig. 3). Skin sensitization without antigen led to significantly decreased eosinophilia in the BAL, and decreased OVA-specific IgE in the serum upon subsequent OVA re-challenge (Supplementary Fig. 2).

### Airway inflammation is antigen-specific

**Figure 4:**
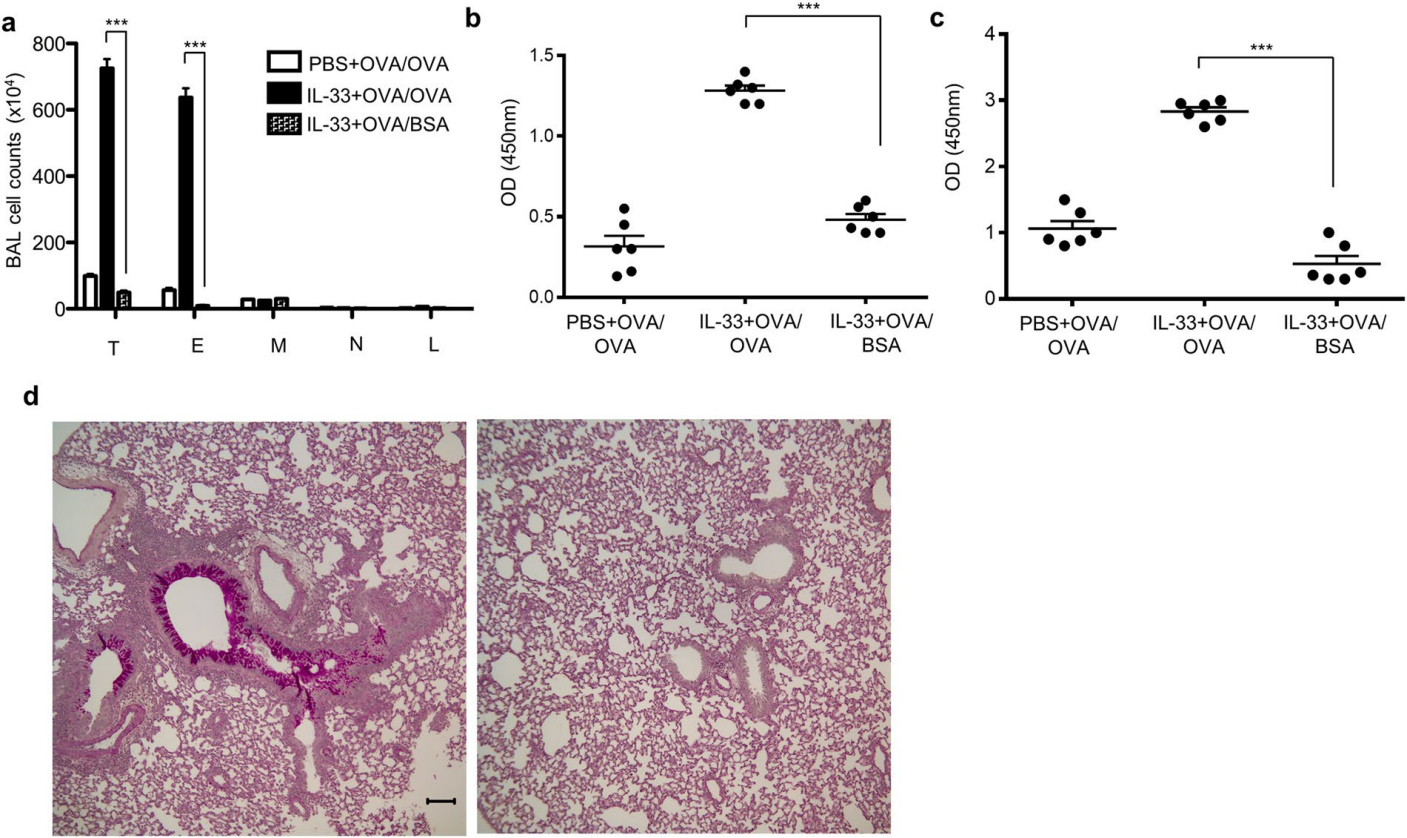
Airway inflammation is antigen-specific. To test the antigen specificity of atopic model, low endotoxin bovine serum albumin (BSA) was used to challenge the mice. (**a**) Cell counts in the BAL fluid. (**b**) OVA-specific IgE in BAL fluid. (**c**) OVA-specific IgE in serum. (**d**) Representative lung tissue cross-sections stained with PAS. Left, IL-33 + OVA/OVA; right, IL-33 + OVA/BSA. Bar, 100 µm. T, total cells; E, eosinophils; M, macrophages; N, Neutrophils; L, lymphocytes. Data were pooled from two independent experiments (n = 6). Error bars indicate the mean ± SD. ***p ≤ 0.001.

To test the antigen specificity of our atopic model, we epicutaneously sensitized mice with IL-33 + OVA, and then challenged in the airways with a second antigen, bovine serum albumin (BSA). In striking contrast, mice that were sensitized to OVA and challenged intranasally with BSA exhibited minimal or no signs of airway inflammation, as characterized by decreased total BAL cellularity, eosinophil counts, antigen-specific IgE responses, and type 2 inflammation in the airway (Fig. 4). Collectively, these data indicate that the effect seen in IL-33+ antigen sensitized and challenged mice was antigen-specific in this model.

### Airway inflammation does not require systemic IL-33

**Figure 5:**
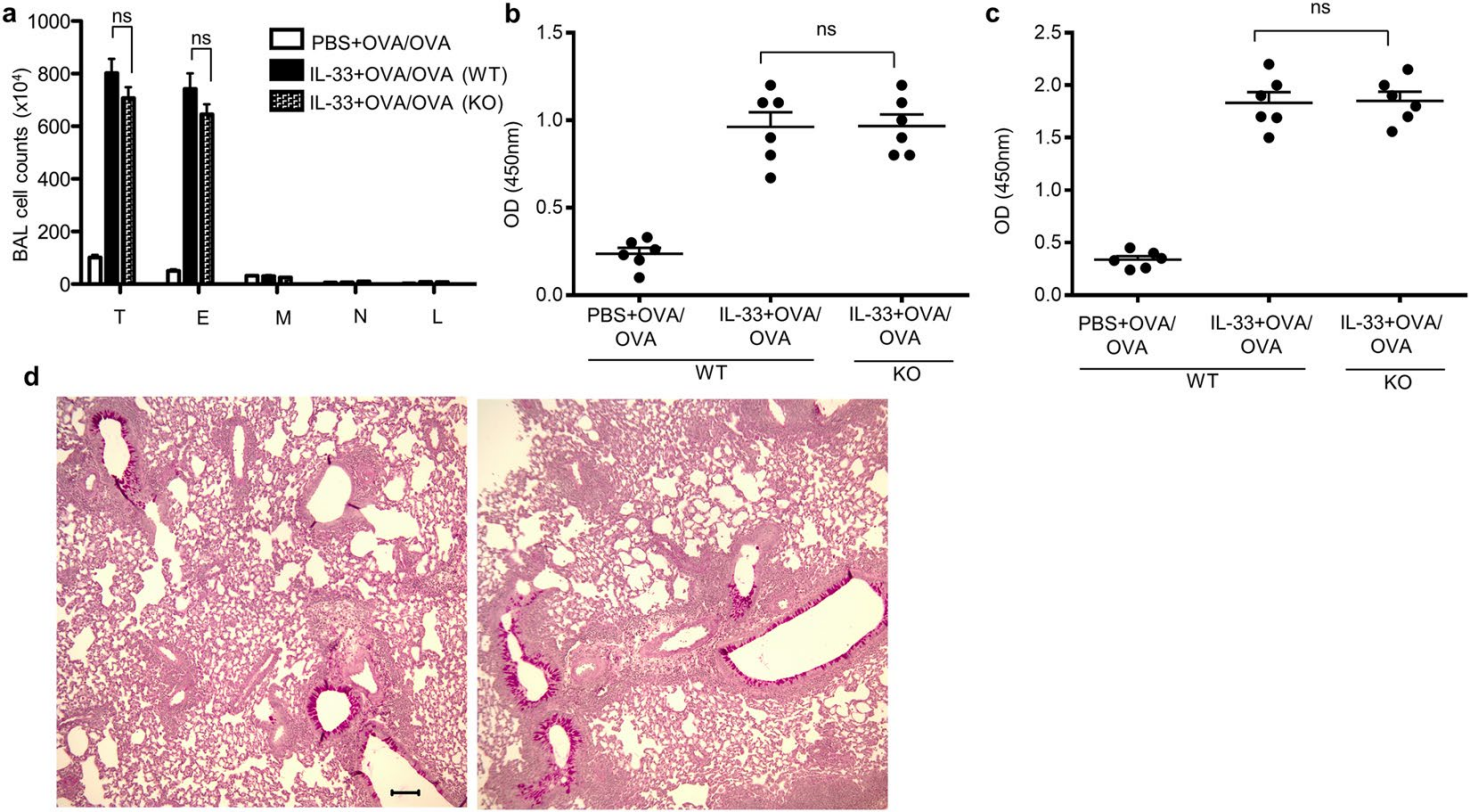
IL-33 is dispensable once skin inflammation develops. *Il33*-deficient (KO) or WT mice treated with PBS or IL-33 plus OVA followed by OVA challenge. (**a**) Cell counts in the BAL fluid. (**b**) OVA-specific IgE in BAL fluid. (**c**) OVA-specific IgE in serum. (**d**) Representative lung tissue cross-sections stained with PAS to visualize mucus-producing goblet cells and airway inflammation. Left, WT; right, *Il33* KO. Bar, 100 µm. T, total cells; E, eosinophils; M, macrophages; N, Neutrophils; L, lymphocytes. Data were pooled from two independent experiments (n = 6). Error bars indicate the mean ± SD. ns, not significant.

IL-33 is known to act on multiple mucosal surfaces, including the skin, the airways and the gastrointestinal tract. Data shown above clearly demonstrates the requirement for IL-33 during sensitization phase to promote allergic responses in the airway on intranasal antigen exposure. To study whether IL-33 is also involved directly during the lung challenge, we first measured circulating IL-33 in the serum on days 14 and 25. IL-33 protein in the serum was undetectable (<15.6 pg/ml; data not shown), suggesting that circulating IL-33 is not required for the aggravated airway responses to antigen when subsequently encountered within the lung in this model. Accordingly, mRNA level of *Il33* in lungs was not increased (data not shown). To further assess the role of IL-33 in this process we took advantage of *Il33*-deficient mice by sensitizing in the skin with IL-33 + OVA as before, and then challenging intranasally with OVA. In this case, the only source of IL-33 is what was injected into the skin during priming. Similar to the WT mice, intranasal antigen exposure in *Il33*-deficient mice resulted in an increased BAL counts, increased antigen-specific serum IgE levels, and accumulation of mucus-producing cells in the airway (Fig. 5). Collectively, these data demonstrate that antigen sensitization in the context of an AD-like skin lesion can promote the development of antigen-induced airway inflammation in the absence of IL-33 during challenge phase.

### Neutralization of ST2 fails to ameliorate TSLP-mediated airway inflammation

**Figure 6:**
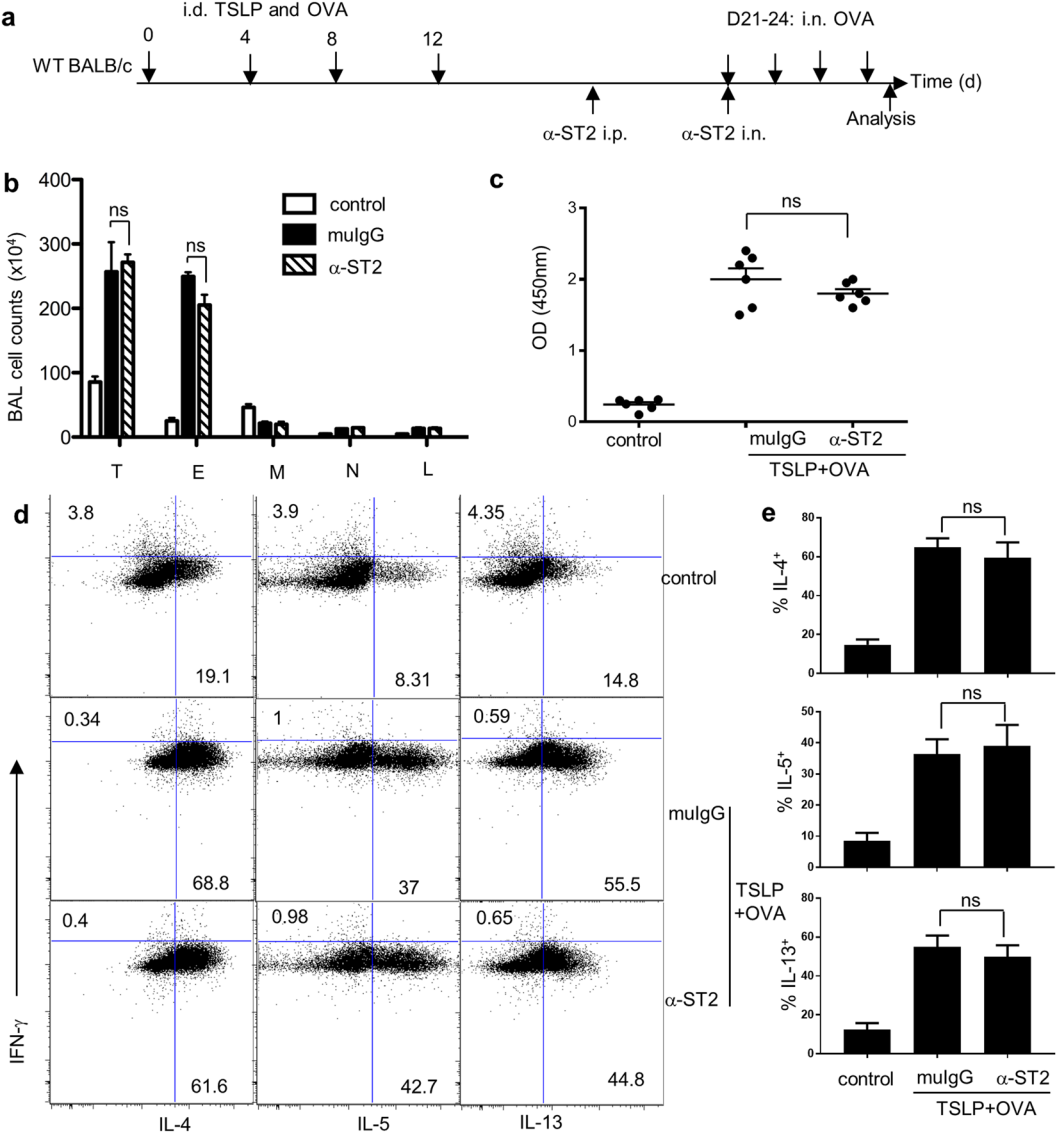
Neutralization of ST2 fails to ameliorate TSLP-mediated airway inflammation. (**a**) Experimental protocol. (**b**) Cell counts in the BAL fluid. (**c**) OVA-specific IgE in the BAL fluid. (**d**) Intracellular cytokine staining of mediastinal lymph node (MedLN) cells. Plots are gated on CD4^+^CD44^hi^ cells. T, total cells; E, eosinophils; M, macrophages; N, Neutrophils; L, lymphocytes. (**e**) Frequency of IL-4^+^, IL-5^+^ and IL-13^+^ in CD4^+^CD44^hi^ cells in MedLN cells. Control, PBS + OVA. Data were pooled from two independent experiments (n = 6). Error bars indicate the mean ± SD. ns, not significant.

The data shown above clearly shows a requirement for IL-33 signaling during the sensitization but not challenge phase in this atopic march model. A question was raised whether this is specific for IL-33-mediated airway inflammation or a general role in a similar set of responses. TSLP is another epithelial cell–derived cytokine linked to allergy; skin sensitization with TSLP leads to the dissemination of allergy from the skin to the lungs^14^. To ascertain whether IL-33 was involved directly during lung challenge in TSLP + OVA-sensitized mice, we examined the effect of blocking ST2 shortly before intranasal OVA challenge (Fig. 6a). As shown in Fig. 6b–e, mice treated with ST2-specific neutralizing mAb responded to intranasal challenge as robustly as isotype control mAb-treated mice, showing that ST2 was not required during airway challenge. In contrast, treatment with anti-ST2 blocked the development of airway inflammation following i.n. administration of IL-33 (data not shown), thus this antibody does effectively neutralize ST2 in the lung. We conclude that IL-33 is not a general therapeutic target to prevent the inflammation within the lung following skin sensitization. Taken together, these data demonstrate that ST2 is dispensable to drive an inflammatory response when antigen is subsequently encountered within the lung.

## Discussion

An understanding of the immunological mechanisms through which antigen sensitization in the skin predisposes to allergic inflammation in airways is crucial for underlying the “atopic march”. IL-33 is a key driver of allergic sensitization in the lungs of newborns, and that IL-33 enhances the function of dendritic cells to induce Th2 cell polarization. HDM administration led to activation of innate lymphoid type 2 cells (ILC2s) in the lungs of newborns in an IL-33-dependent manner^30^. Here we demonstrate that excess of IL-33 in skin leads to an aggravation of a concomitant OVA-induced asthma-like airway inflammation. Our data demonstrate that there are distinct requirements for epithelial cell (EC)-derived cytokines within specific target organs and during the early versus late phase of the allergic inflammatory response.

In the present study, we demonstrated that intradermal administration of IL-33 is crucial for generating allergen sensitization and subsequent development of allergic airway inflammation. IL-33 is an epithelial cell-derived cytokine which signals through ST2, a receptor expressed by T cells, mast cells, DCs, basophils, eosinophils, and ILC2s^29^, and may be a key factor in the establishment of allergic disease in the skin and other sites. Our data exclude that lung locally produced IL-33 could be important for the development of asthma, given that both mRNA and protein levels of IL-33 were not increased in animals. *Il33*-deficient mice responded to the intranasal challenge as robustly as the *Il33*-sufficient mice. The aggravating effect of IL-33 on allergic airway inflammation could be mediated through factors induced locally in the skin and/or systemically in AD mice. Further studies are required to uncover how these factors mediate allergic asthma pathogenesis.

Despite the overlapping signaling pathways of these EC-derived cytokines, our work and others clearly reveal that IL-33, IL-25 and TSLP are differentially required to promote the atopic march^12–17^. The distinct requirements for these cytokines during sensitization and challenge and within different target organs provides a mechanistic basis for the growing clinical recognition of the heterogeneity of allergic diseases at different mucosal sites. During preparation of this manuscript, Galand *et al*. reported that skin IL-33 released through tape stripping enhanced IgE-mediated oral anaphylaxis^31^, which also implicate the skin as an important portal of sensitization in atopic march. ST2 expression by mast cells were important in driving food-induced anaphylaxis, since the disease was attenuated in *Kit* ^*w-sh/w-sh*^ mice which are deficient in mast cells, and transfer of bone marrow-derived mast cells from wild-type but not ST2-deficient mice could restore anaphylaxis in these mice. Tape stripping increases cutaneous expression of both IL-33 and TSLP, but not IL-25. Question remains whether IL-33 is redundant in causing Th2 responses to allergens. Elucidating how these EC-derived cytokines regulate their target cell populations at different sites and during the early and late stages of disease will be important in furthering our understanding of the natural history of atopic diseases, and how interventions that impact these cytokines may differentially influence disease development and progression. Our study provides a plausible explanation of why a great number of AD patients develop asthma and other allergic disorders later in life, suggesting that IL-33 may be a promising target in the prevention of allergic diseases. Our data also indicate that the IL-33/ST2 axis is not involved in the TSLP-mediated model of airway inflammation and that IL-33 is not a general therapeutic target to prevent the allergic asthma once AD develops. Further studies are needed to determine whether targeting IL-33 can treat AD, and whether clinical control of AD with IL-33 blockade will lower the incidence of asthma.

## Materials and Methods

### Ethics approval

All animal experiments in this study were approved by the Institutional Animal Care and Use Committee of Benaroya Research Institute, and were performed in accordance with the approved guidelines for animal experimentation at the Benaroya Research Institute.

### Mice and treatments

6 to 8-wk-old female BALB/c mice were obtained from Charles River Laboratories. *Il1rl1*-deficient mice were provided by Dr. Andrew McKenzie (Medical Research Council Laboratory of Molecular Biology, UK). *Il-33*-deficient mice were provided by Dr. Dirk Smith (Amgen Corp.). All mice were certified to be specific pathogen–free and cared for in accordance with the guidelines of the Institutional Animal Care and Use Committee at Benaroya Research Institute (Seattle, WA). Intradermal injections were performed as previously described^14, 15^. Briefly, 5 µg TSLP (Amgen Corp.) or 2.5 µg IL-33 (R&D Systems and BioLegend) with 2.5 µg OVA (A7642; Sigma-Aldrich) or 2.5 µg HDM (Greer Laboratories) were injected intradermally in a 100 µl volume of sterile PBS every three days for a total of 4 times. Intranasal challenges with 25 µg OVA (A7642; Sigma-Aldrich) or low endotoxin BSA (Sigma-Aldrich) or 10 µg HDM in a total volume of 30 ml PBS were performed as described previously^32, 33^. For blockade with ST2-specific mAb, mice were injected with 500 μg of control mouse IgG1 or muST2-specific muIgG1 mAb (Amgen Corp.) intraperitoneally on days 15 and 19. For i.n. blockade, 50 μg of antibody was used instead.

### Cell culture

Inguinal and mediastinal lymph node cells were isolated and cultured in RPMI medium with 10% fetal calf serum, penicillin, and streptomycin, with 100 μg/ml OVA for 48 hours. Cells were then stimulated with PMA and ionomycin in the presence of brefeldin A for 4–5 h. The cells were stained and analyzed for cytokine production by flow cytometry. Further analyses were performed using FlowJo software (Tree Star, Inc.).

### Bronchial alveolar lavage, tissue fixation and staining

BAL was performed 24 hours after the last aerosol challenge as described previously^32, 33^. BAL cells were resuspended in PBS containing 1% BSA and counted using a hemocytometer. Differential cell counts were performed on cytospin cell preparations stained with a modified Wright-Geimsa stain (Diff-Quiktm Stain Set, Siemens). After BAL extraction, lungs were excised completely from the chest cavity, inflated with 10% neutral buffered formalin (Fisher BioTech) and fixed at room temperature overnight in the same solution. Tissues were embedded in paraffin, sectioned and stained with periodic acid Schiff (PAS). Images were acquired using Leica DM2500 Upright microscope with Insight 4 megapixel color CCD camera (Diagnostic Instruments).

### ELISA

Detection of antigen-specific IgE has been described before^32, 34^.

### Statistics

Statistical analyses were performed with Prism software (GraphPad). Two means were compared using nonparametric 2-tailed Mann-Whitney test. Three or more means were compared using one-way ANOVA with a Tukey post-hoc test with significance between groups represented as * for p ≤ 0.05, ** for p ≤ 0.01, and *** for p ≤ 0.001.

## Electronic supplementary material

Supplementary Information
