# Supplementary material for: Intradermal administration of IL-33 induces allergic airway inflammation
Source: Sci Rep. 2017 May 10;7:1706. doi: 10.1038/s41598-017-01863-5 (PMC5431780; doi:10.1038/s41598-017-01863-5)
Supplement: Supplementary file 1 — Supplementary Information [file 41598_2017_1863_MOESM1_ESM.pdf]

Intradermal administration of IL-33 induces allergic airway inflammation

Hongwei Han[1] and Steven F. Ziegler[1,2]

[1] Immunology Program, Benaroya Research Institute, Seattle, Washington 98101, USA

[2] Department of Immunology, University of Washington School of Medicine,
Seattle, Washington 98195, USA

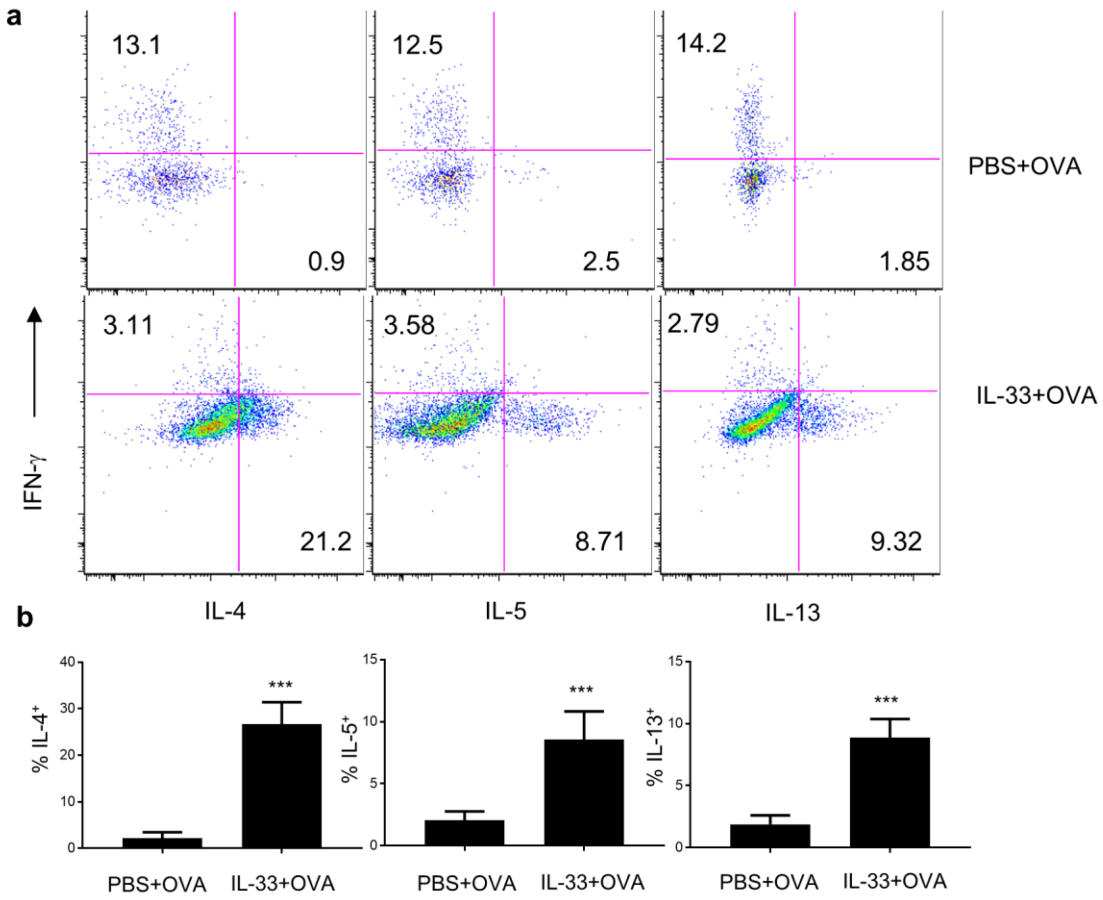

Supplemental Figure 1. Th2 polarization in the lung lymph nodes. (a) Intracellular cytokine staining of mediastinal lymph node (MedLN) cells. Plots are gated on $CD4^{+}CD44^{hi}$ cells. (b) Frequency of $IL\text{-}4^{+}$, $IL\text{-}5^{+}$ and $IL\text{-}13^{+}$ in $CD4^{+}CD44^{hi}$ cells in MedLN cells. Control, PBS+OVA. Data were pooled from two independent experiments (n=6). Error bars indicate the mean ± SD. *** p≤ .001.

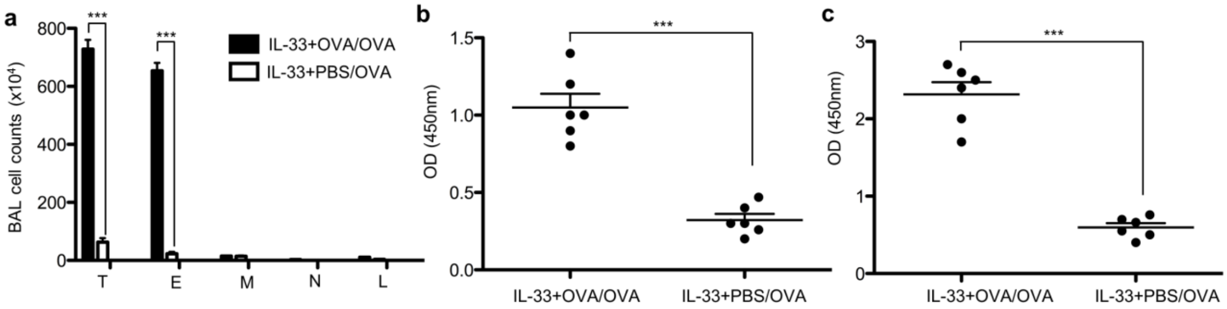

Supplemental Figure 2. Skin sensitization without antigen results in significantly reduced airway inflammation. (a) Cell counts in the BAL fluid. (b) OVA-specific IgE in BAL fluid. (c) OVA-specific IgE in serum. T, total cells; E, eosinophils; M, macrophages; N, Neutrophils; L, lymphocytes. Data were pooled from two independent experiments (n=6). Error bars indicate the mean ± SD. *** p≤ .001.
